# Nodal degree centrality in the default mode-like network of the TgF344-AD Alzheimer’s disease rat model as a measure of early network alterations

**DOI:** 10.1038/s41514-024-00151-7

**Published:** 2024-06-20

**Authors:** Saba Amiri, Monica van den Berg, Mohammad-Reza Nazem-Zadeh, Marleen Verhoye, Mahmood Amiri, Georgios A. Keliris

**Affiliations:** 1 Neuroscience Research Center, Shahid Beheshti University of Medical Sciences, Tehran, Iran; 2 Bio-Imaging Lab, University of Antwerp, Antwerp, Belgium; 3 µNEURO Research Centre of Excellence, University of Antwerp, Antwerp, Belgium; 4 Research Center for Molecular and Cellular Imaging, Tehran University of Medical Sciences, Tehran, Iran; 5 Department of neuroscience, Monash university, Melbourne, Vic, Australia; 6 Medical Technology Research Center, Institute of Health Technology, Kermanshah University of Medical Sciences, Kermanshah, Iran; 7 Institute of Computer Science, Hellas Foundation for Research & Technology - Hellas, Heraklion, Crete, Greece

**Keywords:** Neuroscience, Diseases

## Abstract

This study investigates brain network alterations in the default mode-like network (DMLN) at early stages of disease progression in a rat model of Alzheimer’s disease (AD) with application in the development of early diagnostic biomarkers of AD in translational studies. Thirteen male TgF344-AD (TG) rats, and eleven male wild-types (WT) littermates underwent longitudinal resting-state fMRI at the age of 4 and 6 months (pre and early-plaque stages of AD). Alterations in connectivity within DMLN were characterized by calculating the nodal degree (ND), a graph theoretical measure of centrality. The ND values of the left CA2 subregion of the hippocampus was found to be significantly lower in the 4-month-old TG cohort compared to the age-matched WT littermates. Moreover, a lower ND value (hypo-connectivity) was observed in the right prelimbic cortex (prL) and basal forebrain in the 6-month-old TG cohort, compared to the same age WT cohort. Indeed, the ND pattern in the DMLN in both TG and WT cohorts showed significant differences across the two time points that represent pre-plaque and early plaque stages of disease progression. Our findings indicate that lower nodal degree (hypo-connectivity) in the left CA2 in the pre-plaque stage of AD and hypo-connectivity between the basal forebrain and the DMLN regions in the early-plaque stage demonstrated differences in comparison to healthy controls. These results suggest that a graph-theoretical measure such as the nodal degree, can characterize brain networks and improve our insights into the mechanisms underlying Alzheimer’s disease.

## Introduction

Alzheimer’s disease (AD) is a neurodegenerative disorder, characterized by the accumulation of amyloid-beta (Aβ) plaques, formation of neurofibrillary tangles, synaptic dysfunction, and neuronal loss that can affect memory, cognitive function, and behavior^1–3^. Although numerous studies and clinical trials have been searching for a better understanding of the pathological mechanisms of AD, disease-modifying treatments are still lacking.

Among a range of neuroimaging tools able to provide more insights into the neuropathological mechanisms, diagnosis, and prognosis of AD, resting-state functional magnetic resonance imaging (rsfMRI) and functional connectivity (FC) have emerged as particularly useful in the early diagnosis and preclinical signs of AD^4,5^. RsfMRI is a noninvasive neuroimaging tool that can be used to extract the FC matrix. Indeed, FC is defined as the association between brain regions reflected in the temporal correlation of their activity measured by using the blood-oxygen-level-dependent (BOLD) signal^6,7^. Thus, FC is considered as an indirect measure of trans-synaptic activity^8^.

Altered FC has been observed in brain regions that are essential to learning and memory, even before appreciable plaque deposition and the onset of clinical symptoms^9,10^. Differences in FC in patients with AD are thought to reflect disruption of brain networks, particularly in regions with amyloid deposition. Altered FC of the default mode network (DMN) in humans is an established feature of AD development, especially at its early stages such as in mild cognitive impairment (MCI) patients, however, the directionality of changes varies^11,12^. The DMN is a large-scale brain network involved in memory consolidation, self-oriented processes, and introspection^11–13^. Structural atrophy, glucose metabolism, brain activity, and functional connectivity studies in AD consistently suggest an increasing disruption in the DMN^14,15^. FC within the DMN also decreases with normal aging, with accelerated decreases in AD^12^. Previous research has reported decreased and increased DMN connectivity as a function of increased amyloid deposition in cognitively healthy older adults^16–18^. Similar to the DMN in humans, a default mode-like network (DMLN) has been observed in macaque monkeys, rats, and mice^19–21^. Indeed, the functions of both DMLN in rats and DMN in humans are spatially similar as they consist of the anterior and posterior cingulate-, orbitofrontal-, temporal-, parietal-, cortices and hippocampus in both species^18,20,22–24^. In addition, a number of recent studies demonstrate that rsfMRI studies looking into brain networks such as the DMLN in rodent models of AD, can provide new insights into how brain networks are affected by the disease^25–28^.

Using histological analysis, previous studies in the TgF344-AD rat model have demonstrated that Aβ plaques and pTau start accumulating with an age dependent gradient from 6 months onwards^29,30^. The goal of this study is to investigate how DMLN connectivity is altered before Aβ plaques are present in the brain, during the so-called pre-plaque stages of AD. The TgF344-AD rat model of AD bears the human APPswe gene and PS1 mutation, which results in the accumulation of amyloid-beta plaques in the brain from 6 months onward. At 4 months of age, no amyloid plaques have been observed in the cortex and hippocampus of TgF344-AD rats, suggesting that this time point qualifies as the pre-plaque stage^9^. Indeed, the soluble amyloid monomers and oligomers have been demonstrated to be present in the brain tissue^9^, which were also detected in the blood of TgF344-AD rats^31^. In addition to amyloid pathology, TgF344-AD rats display accumulation of hyperphosphorylated tau, a precursor of tau tangles, from 6 months onward^9^.

Recent studies based on the graph theory have indicated brain network alterations in patients with AD^32–34^. Graph theory has been used in AD to reveal compensatory mechanisms in MCI patients and cognitively healthy adults^35^. Nevertheless, we aim to investigate potential alterations in the DMLN during the pre-plaque and early-plaque stages of AD in the TgF344-AD rat model. To this end, we use graph-theoretical network analysis^36–38^. In graph theory, the brain networks can be described by a cluster of nodes and edges that represents brain regions and their structural or functional connectivity^36,37^. Specifically, centrality metrics, can determine the importance of each node in a brain network and this makes them appropriate measures to capture the complexity of functional connectivity. Among these metrics, the nodal degree (ND), i.e., the number of connections a node has with other nodes in the network, is one of the most popular measures of centrality and it is directly calculated from the functional connectivity matrix. Furthermore, it has a high correlation with other centrality metrics (betweenness centrality, clustering coefficient, node neighbor’s degree, and closeness centrality)^39–42^. In this study, we estimated the ND as a function of the density of connections in the FC matrix (a density of x% means that only the edges with x% highest FC were used) to compare the FC in DMLN between the TgF344-AD and wild-type (WT) rats at 4 and 6 months of age that represent pre- and early-plaque stages of disease progression.

## Results

### Identifying DMLN regions in the dataset

**Fig. 1:**
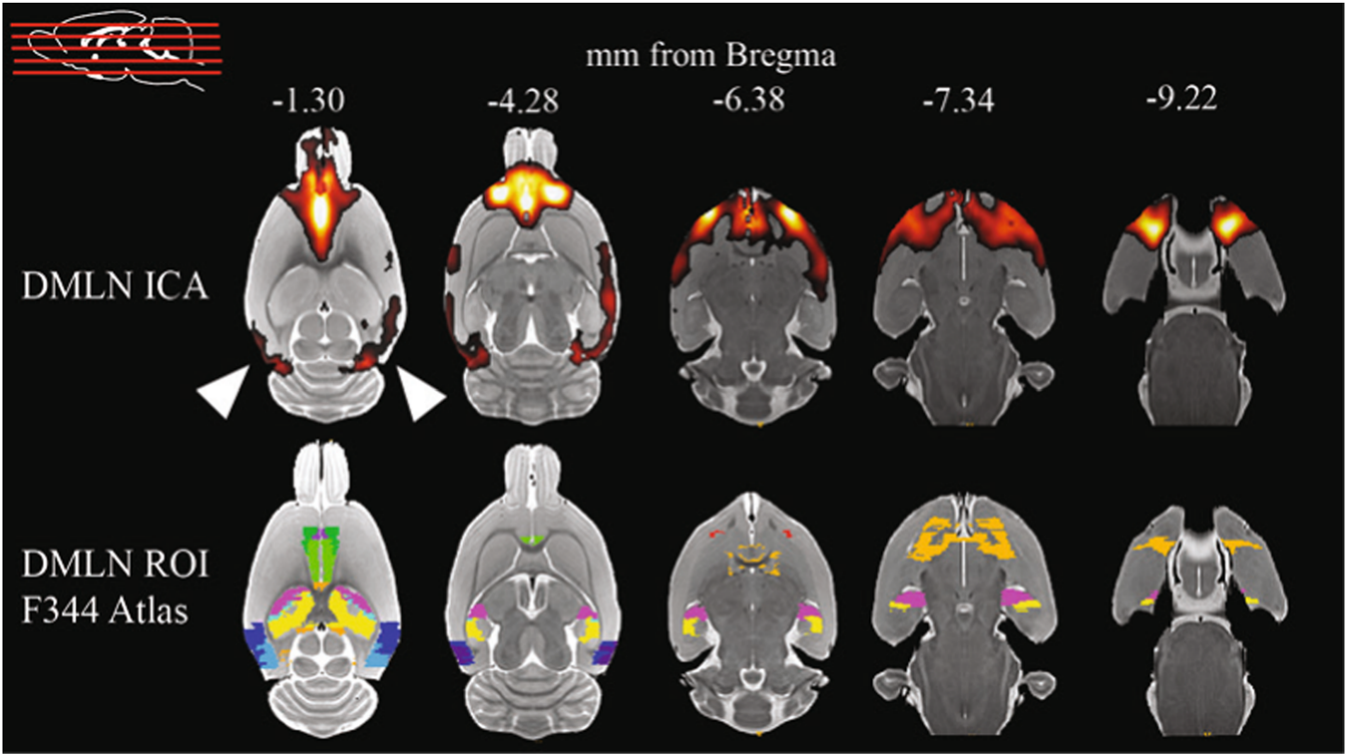
Independent component analysis (ICA) and atlas regions of interest (ROIs). ICA reveals a component that encompasses major nodes of the DMLN in the dataset (top row). The visual cortex (arrowheads) is functionally connected in the DMLN component. The bottom row shows the ROIs of the DMLN and hippocampus which were used in further analysis based on theF344 anatomical rat brain atlas. Each region has a distinct color.

Independent component analysis is a data-driven method to construct spatial correlation maps from the voxel-based time-series. This analysis resulted in a component which encompasses the majority of the DMLN regions in rats, including the cingulate cortices, retrosplenial cortex, orbitofrontal cortex, prelimbic cortex, basal forebrain, temporal association cortex and parietal cortex. Interestingly, the visual cortex was also included in this component (Fig. 1), suggesting that the visual areas (V1 and V2) are also part of the DMLN in this dataset. The hippocampal regions do not show up on the ICA spatial map, however these regions were included in the analyses (CA1, CA2, CA3), given the importance of this brain structure in AD.

### Differences in the ND values between TG_4M and WT_4M cohorts (pre-plaque stage)

**Fig. 2:**
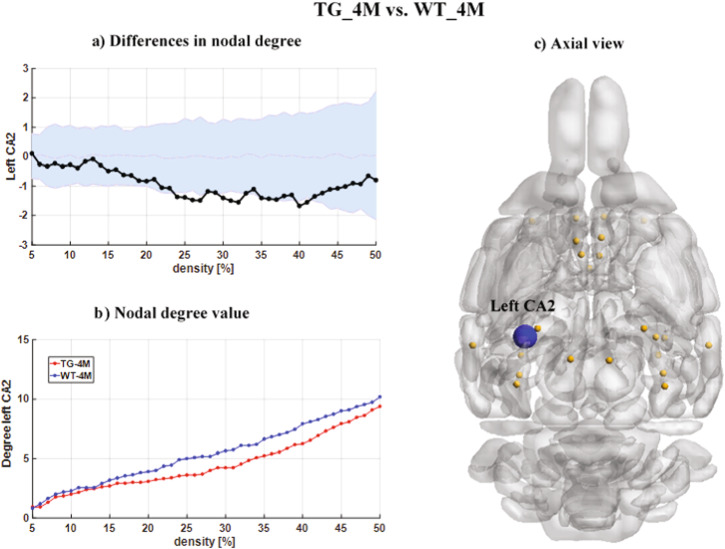
Nodal degree differences between the TG_4M and WT_4M cohorts for DMLN. **a** The nodal degree differences (statistically significant, *p* < 0.05, non-parametric permutation test, FDR corrected) between TG_4M and WT_4M cohorts in the DMLN. The black points which show the actual nodal degree differences between TG_4M and WT_4M cohorts are significant (*p* < 0.05, non-parametric permutation test, FDR corrected) if they fall outside the confidence intervals (light blue zone). **b** The nodal degree value as a function of network density. Blue and red points indicate the nodal degree values for WT_4M and TG_4M, respectively. **c** The schematic shows an axial view of the DMLN with network nodes in orange and the hypo-connected left CA2node in blue.CA2: subregion of the hippocampus.

We compared the differences in the ND values at 4 months of age, before formation of the amyloid plaques in the brain. Statistical comparisons across the 25 selected DMLN areas including hippocampus^24^ identified significant changes in one region of interest. The ND values in the TG_4M rats was decreased relative to the WT_4M littermates in the left CA2 subregion of the hippocampus. Figure 2a demonstrates the differences in ND (negative values denote decreases in the ND value of the TGs) for different densities of connections in the FCM along with the confidence intervals estimated from the < (see Materials and Methods). Black points illustrate the actual difference in ND values between the TG_4M and WT_4M cohorts, and confidence intervals presented by the light-blue zone. The actual difference value (black points) is significant (*p* < 0.05, non-parametric permutation test, FDR corrected) if it falls outside the confidence intervals (light-blue zone). We observed that significant values were found for densities ranging from 24% to 41%. Figure 2b shows the ND values as a function of connection density in the DMLN areas (in the Supplementary Table 1, we have provided the detailed p-values after FDR correction for each density in different brain regions).

### Differences in the ND values between TG_6M and WT_6M cohorts (early plaque stage)

**Fig. 3:**
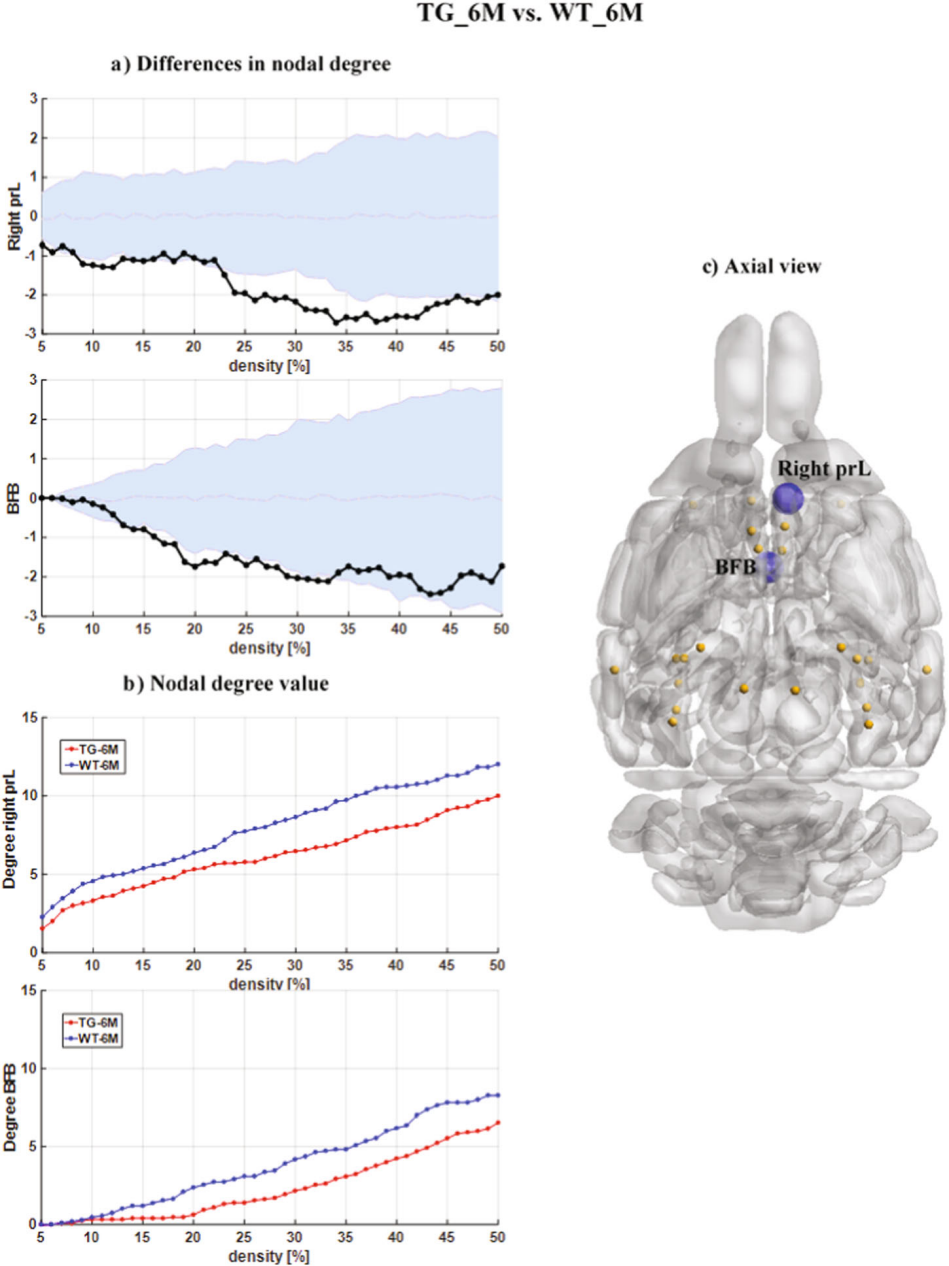
Nodal degree differences between the TG_6M and WT_6M cohorts for DMLN. **a** The nodal degree differences (statistically significant, *p* < 0.05, non-parametric permutation test, FDR corrected) between TG_6M and WT_6M cohorts in the DMLN. The black points represent the actual nodal degree difference between TG_6M and WT_6M cohorts. The actual difference value (black color points) is significant (*p* < 0.05, non-parametric permutation test, FDR corrected)if it falls outside the confidence intervals (light blue zone). **b** The nodal degree value as a function of network density. Blue and red points indicate the nodal degree values for WT_6M and TG_6M, respectively. **c** The schematic shows an axial view of the DMLN with network nodes in orange and the hypo-connected BFB and right prL nodes in blue. prL: prelimbic Cortex, BFB: basal forebrain.

Similar analysis was then performed across the two cohorts at 6 months of age. Differences were observed in the right prelimbic cortex (prL) and basal forebrain (BFB) as shown in Fig. 3a. In both regions, we observed decreases in the ND values in the TG_6 M cohort compared to the WT_6M littermates. Specifically, in the right prelimbic cortex, the ND differences were significant across almost all densities.

### Changes in the ND values with time across the TG and WT cohorts

**Fig. 4:**
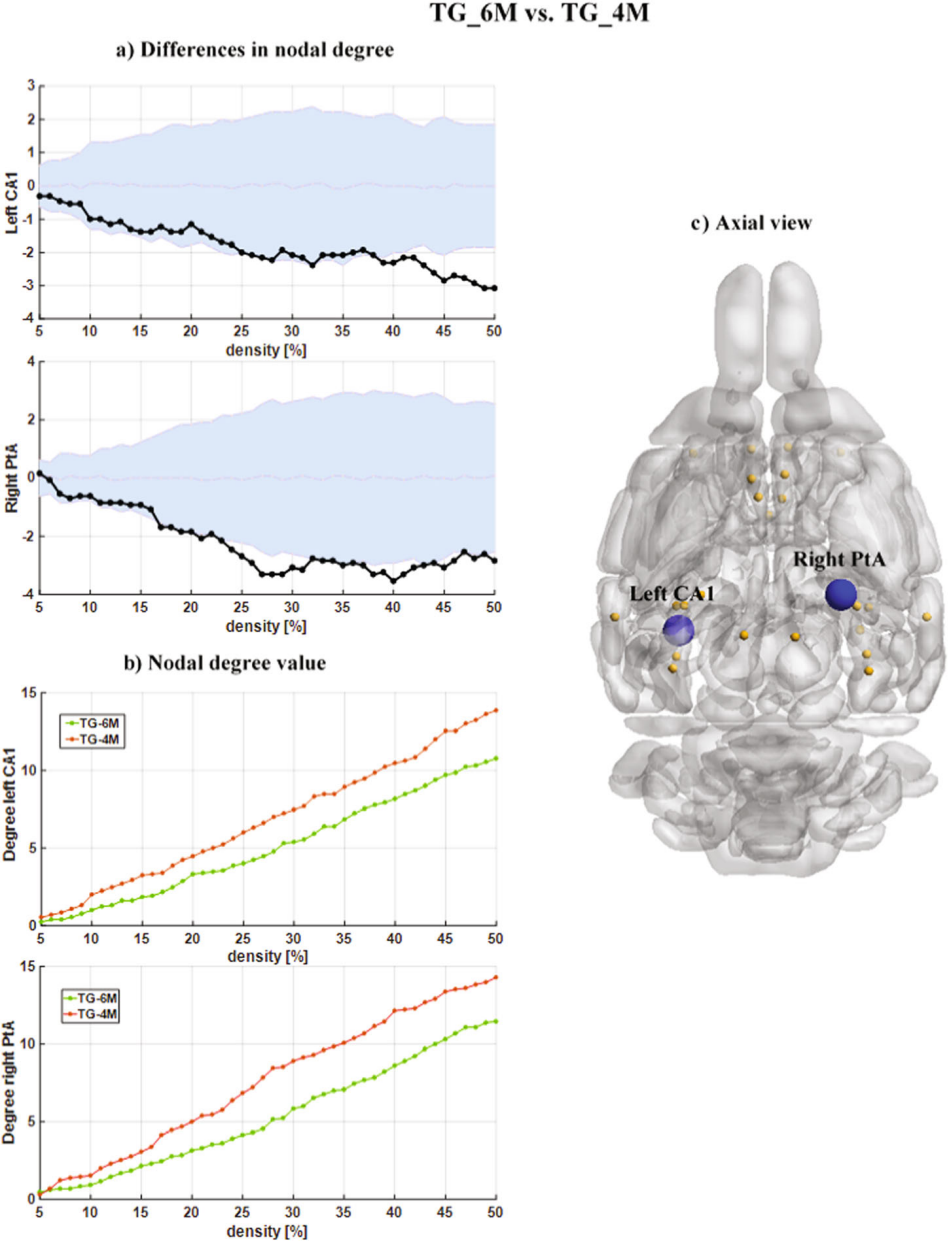
Nodal degree differences between the TG_6M and TG_4M cohorts for DMLN. **a** The nodal degree differences (statistically significant, *p* < 0.05, non-parametric permutation test, FDR corrected) between the TG_6M and TG_4M cohorts for DMLN. The black points represent the actual nodal degree difference between the TG_6M and TG_4M cohorts. The actual difference value (black color points) is significant (*p* < 0.05, non-parametric permutation test, FDR corrected) if it falls outside the confidence intervals (light blue zone). **b** The nodal degree value as a function of network density. Green and brown points indicate the nodal degree values for TG_6M and TG_4 M, respectively. **c** The schematic shows axial view of the DMLN with network nodes in orange and the hypo-connected left CA1 and right PtA nodes in blue. CA1 subregion of the hippocampus, PtA parietal association cortex.

**Fig. 5:**
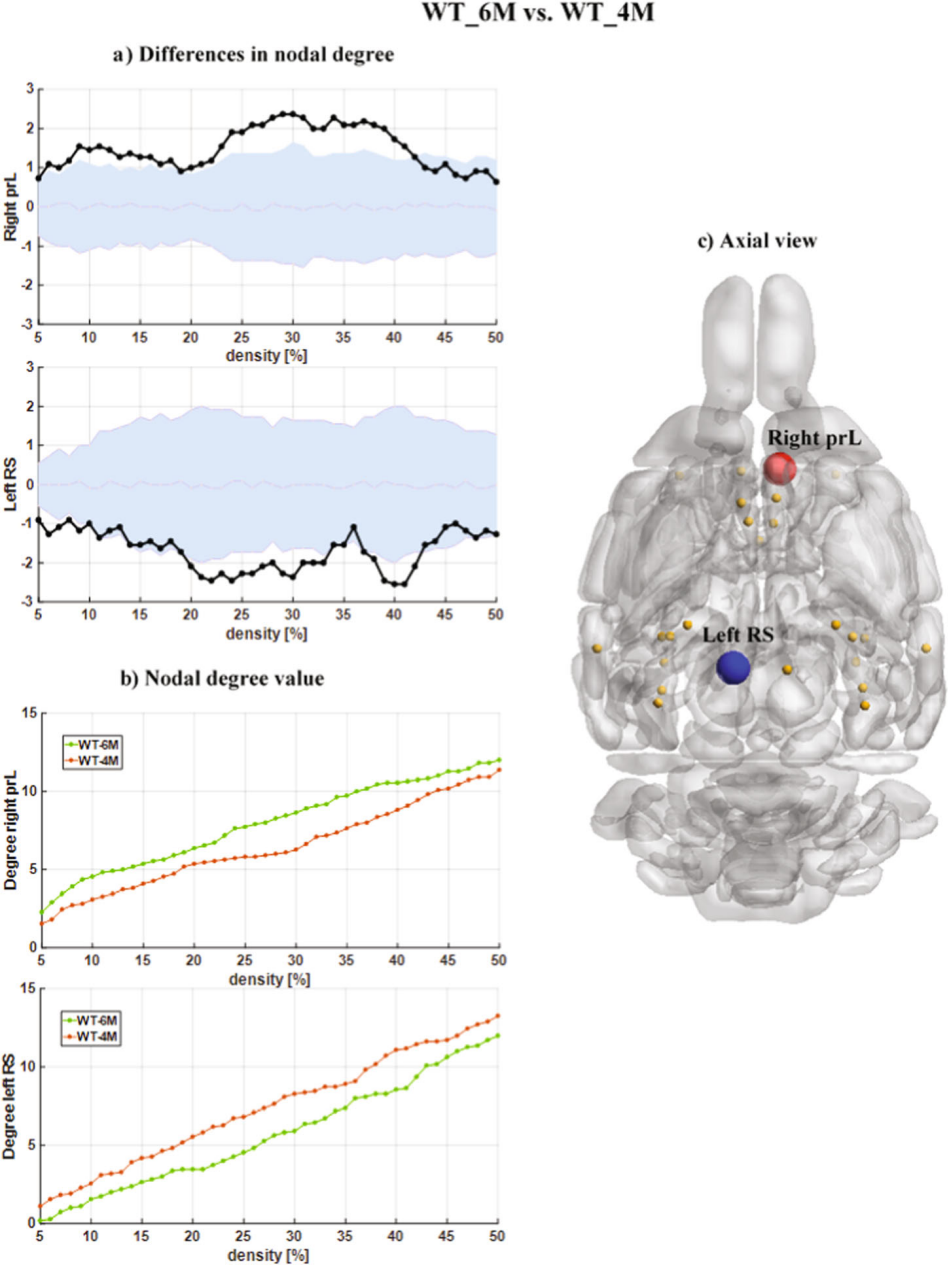
Nodal degree differences between the WT_6M and W_4M cohorts for DMLN. **a** The nodal degree differences (statistically significant, *p* < 0.05, non-parametric permutation test, FDR corrected) between WT_6M and W_4M cohorts for DMLN. The black points represent the actual nodal degree difference between WT_6M and WT_4M cohorts. The actual difference value(black color points) is significant (*p* < 0.05, non-parametric permutation test, FDR corrected) if it falls outside the confidence intervals (light blue zone). **b** The nodal degree value as a function of network density. Green and brown points indicate the nodal degree values for WT_6M and WT_4 M, respectively. **c** The schematic shows axial view of the DMLN with network nodes in orange and the hypo-connected left RS node in blue and hyper-connected right prL node in red.prL: prelimbic cortex, RS: retrosplenial cortex.

To better understand the developmental effects on the ND differences across the two cohorts with age, we then compared the ND values of the same group across two time points (6M vs 4 M). Indeed, the aging effect and growing up from 4-month to 6-month were explored in the TG and WT groups, separately. In the TG cohort, we found significant decreases of ND values (Fig. 4). Compared to the TG_4M, the TG_6M subjects showed significantly lower ND values in the left hippocampal CA1 and right parietal association cortex (ptA). In the WT_6M subjects, we observed an increase in the ND values in the right prelimbic cortex (prL) and a decrease in the left retrosplenial (RS) compared to the TG_4M (Fig. 5). Notably, increases and decreases in the ND values with age in the WT cohort cover a wider range of densities, starting from D = 5%, when the strongest connections are only taken into account. (in the Supplementary Table 1, we have provided detailed p-values after FDR correction for each density in different brain regions).

## Discussion

In this study, we investigated the connectivity of DMLN regions in the TgF344-AD rat model of AD compared to age and gender matched WT littermates. To this end, we calculated a graph theoretical measure, namely the ND centrality, to characterize connectivity of 25 selected DMLN regions of interest. Furthermore, we assessed the effects of age by following the longitudinal changes of ND values as the rats grew up from 4-month (pre-plaque stage of AD) to 6-month (early-plaque stage of AD) in both cohorts. Our results showed a significantly decreased ND values (hypo-connectivity) in the 4-month-old TG rats (TG_4M) in the left CA2 subregion of the hippocampus compared to the 4-month-old WT rats (WT_4M). Compared to the WT_6M, we found decreased ND values in the TG_6M in the basal forebrain (BFB) and right prelimbic cortex (prL). Furthermore, our results showed that in both TG and WT rat cohorts, that ND values in the DMLN regions change as the rats getting mature and this should be considered when trying to identify biomarkers.

In this paper we found that ND values of the left CA2 was significantly lower in the TG_4M rats compared to the WT_4M group. This indicates a lower ability in local processing and a lower distributed interaction with other nodes within the network. Recent evidence suggests that CA2 has been strongly associated with social behaviors, including social memory, stress response, and pattern separation^43,44^. Some studies in TgF344-AD rats have demonstrated that impairments in spatial memory occur at 4 months of age^45–47^ which might be the result of the decreased ND values of the CA2 observed in the current study. However, future experiments that perform rsfMRI and behavioral analysis are necessary to evaluate if changes in behavior could be correlated to the changes observed in rsfMRI. The physiological and anatomical properties of CA2 pyramidal cells establish this region as a central computational hub within the hippocampus, playing a crucial role in information processing in the hippocampus^44^. Many studies indicated early stages of AD are characterized by hypo- or hyper-functional connectivity between the hippocampus and other brain areas^48–50^. It has been shown that there is hypo-connectivity between the hippocampus and DMN regions in both AD and MCI cases^48–50^. During the pre-plaque stage of AD, it appears that the information processing function of the hippocampus is disrupted.

Consistent with recent reports, we observed a decreased ND in the basal forebrain in TG_6M rats compared to the WT_6M rats at the early-plaque stage. Basal forebrain cholinergic system, which provides acetylcholine to various brain regions, including the DMN, is particularly affected in Alzheimer’s disease and MCI^51,52^. This disruption in cholinergic transmission can lead to the impaired communication between the basal forebrain and the DMN, contributing to cognitive deficits^51,52^. A recent study in patients with subjective cognitive decline showed a significant decrease in functional connectivity between the basal forebrain and the hippocampus^53^. Furthermore, another study found strong associations between basal forebrain connectivity, cerebrospinal fluid biomarkers, and blood serum biomarkers of Alzheimer’s disease in patients at various stages of the disease^54^. A recent study utilizing the same dataset demonstrated that the disconnection between the basal forebrain and the DMLN regions during quasi-periodic patterns could potentially serve as a biomarker for Alzheimer’s disease^9^.

In this paper we showed that the ND values of the right prelimbic cortex was significantly lower in the TG_6M rats compared to the WT_6M group. The prelimbic cortex is part of the prefrontal cortex, which plays a crucial role in cognitive processes, including working memory and attention^55^. In AD, alterations in functional connectivity of the prefrontal cortex and DMN regions have been reported. Indeed, previous studies in AD patients showed hypo-connectivity in FC between the prefrontal cortex and the hippocampus^56,57^.

Our results also indicate a significant decrease in ND values in both the left CA1 region of the hippocampus and the right parietal association cortex as the age of the TG subjects increased. The CA1 region of the hippocampus is an important subfield involved in memory formation and retrieval^58^. Studies have shown disrupted functional connectivity between the CA1 region and the other DMN regions in individuals with AD^18,59^. This disruption may contribute to cognitive deficits and memory impairments, the characteristic of the disease^18,60^. Consistent with previous reports, we found hypo-connectivity in hippocampal CA1 in 6-month-old TG, compared to the 4-month-old TG. The hippocampus has a key role in many forms of learning and memory^18,59^. MCI subjects initially experiencing hyperactivity in the hippocampus at baseline, developed a hippocampal hypoactivity over time. Particularly, the rate of the decrease in hippocampal activity was shown to be correlated with the rate of decline in memory performance^18,60^. Similar results have been observed in rsfMRI studies in mice, soluble Aβ, in the pre-plaque stage, caused hyper-connectivity in the hippocampus. In contrast, an overall decrease in the network FC was observed after the onset of plaques^25,26,61^. Consistent with these studies, we found decreased ND in hippocampal CA1 in the early-plaque stage (TG_6M), compared to the pre-plaque stage (TG_4M).

We observed decreased ND in the left retrosplenial cortex and increased ND in the right prelimbic cortex in WT_6M, compared to the WT_4M. Several studies have reported a decreased functional connectivity of the retrosplenial cortex within the DMN with increased age^62–64^. This decline in connectivity may be associated with age-related cognitive decline, particularly in memory and spatial navigation processes that involve the retrosplenial cortex^62^. Furthermore showed that normal aging in mice is associated with declines in functional connectivity in the retrosplenial cortex^64^. While these findings are not specific to the prelimbic cortex, some studies have reported increased connectivity within the prefrontal cortex with advancing age. This heightened connectivity may reflect compensatory mechanisms or adaptations in response to age-related changes in the brain. It could potentially serve as a neural compensation strategy to maintain cognitive function.

The aim of the current study was to identify alterations in brain connectivity before cognitive symptoms arise, since cognitive symptoms in human AD patients are only present during relatively advanced stages, when irreversible damage is already inflicted to the brain. Numerous studies have demonstrated that amyloid depositions are poorly correlated with cognitive deficits in humans, which is also a major drawback of the amyloid cascade hypothesis^65^. However, TgF344-AD rats, as well as other mouse models of AD, have been demonstrated to display cognitive symptoms already at very early stages of AD, before amyloid depositions are present in the brain^66,67^. A large number of studies have focused on the behavioral phenotype of the TgF344-AD rats. Cognitive deficits have been reported in TgF344-AD rats from 6 months onward^29,68,69^. Two studies from two different facilities have observed altered spatial navigation and spatial memory in 4-month-old TgF344-AD rats using frequently used behavioral tests (e.g., Barnes Maze and Active Allothetic Place Avoidance test)^45,46^. Another research group observed impairments in working memory in 5-month-old TgF344-AD rats, but not at later time points^47^. Aforementioned results demonstrate the variability in behavioral outcomes in these rats, which are in part the result from different behavioral paradigms used. A consistent behavioral outcome is increased anxiety in TgF344-AD rats from 4-months onward^70,71^.

The majority of the preclinical MRI studies in rodents use anesthesia to limit stress and motion. The DMLN is one of the networks which is robustly observed under different anesthesia protocols and under different levels of anesthesia^72,73^. Moreover, several studies have attempted to compare the human DMN and the rodent DMLN and observed high spatial similarity^74^. Previous studies have tried to evaluate the interaction between attention networks and the DMLN in anesthetized rats^75^. DMLN connectivity has been demonstrated to be suppressed upon visual stimulation. In addition, cholinergic stimulation also induced decreased FC within the DMLN^24^. Similar observations have been done in humans, where DMN activity and connectivity are suppressed upon initiation of a task, suggesting high homology between the human DMN and rodent DMLN, even under anesthesia.

In conclusion, we demonstrated that the default mode-like network is functionally impaired, characterized by hypo-connectivity between its constructing brain regions, especially in the left CA2 subregion of the hippocampus, even at the pre-plaque stage of Alzheimer’s disease. Our findings suggest that the hypo-connectivity between the basal forebrain and the DMLN regions and hypo-connectivity of prelimbic cortex in early-plaque stage could potentially serve as an early biomarker for Alzheimer’s disease. Furthermore, our study demonstrates the ability of nodal degree to characterize the brain functional connectivity and provide invaluable information towards the development of biomarkers capable of early diagnosis of Alzheimer’s disease and improving our insights into its underlying mechanisms.

### Limitations and future directions

It would be interesting to investigate if changes in ND values could be correlated to changes in cognition. However, the current study did not include behavioral assessment. Indeed, if future fMRI studies would include behavioral assessment to demonstrate if alterations in connectivity and/or ND values are correlated with cognitive impairments. We would like to indicate that although this study provides invaluable insights into the changes in the DMLN connectivity at very early stages of disease in an excellent rat model, which provides all the hallmarks of AD, this study does not claim to have identified biomarkers. To that end, one should design experiments with larger samples that is an essential requirement for more definitive and trustworthy results for diagnostic and therapeutic purposes.

The current paper only included male TgF344-AD rats to limit the variability and the number of animals used in this study since previous FC studies observed differences in the static FC between genders. In humans, studies have demonstrated that disease progression is faster in women compared to men^76^. This sex effect is also observed in TgF344-AD rats. A recent study in TgF344-AD rats showed that amyloid pathology and neuro-inflammation are more severe in females. Nonetheless, cognitive and behavioral deficits were found to be less severe in female rats compared to male rats^77^. Moreover, an electrophysiology study demonstrated that the onset of synaptic dysfunction in the hippocampal circuit occurs later in females (9 M) compared to males (6 M), possibly due to the neuroprotective effects of estrogen^78^, demonstrating that gender differences are also present in TgF344-AD rats. Future studies should be including female TgF344-AD rats to validate the current findings, and to improve translation to humans.

Our previous study showed that at 4 months of age, soluble amyloid species were increased, in the absence of amyloid plaques^9^. At 6 months of age, amyloid plaques were observed in the cortex and hippocampus of all TgF344-AD rats. Moreover, hyperphosphorylated tau was observed in the locus coeruleus at 6 months of age, as was reported before in literature^30^. In addition, transient neuro-inflammation was observed in the nucleus basalis of Meynert at 4 months of age, but not at 6 months of age. However, because of the ex-vivo nature of the histological analysis, hence our use of a separate cohort for the histological analyses, it is not possible to correlate the current findings to these histological outcomes. Future studies on the correlation between histopathology and changes in ND would be valuable.

The focus of this paper was the use of graph theory metrics as an early sign of network alterations in AD. We hypothesized that functional alterations would precede structural changes, as was observed in the previous structural MRI study^10^. This study demonstrated no age-dependent genotypic-specific changes in the volume of specific brain regions, which would have been indicative of potential cortical atrophy. The ages included in the current study match the first timepoint of the study of Anckaerts et al.^10^. Therefore, we did not investigate if anatomical differences were present between groups. In addition, a technical and interpretational limitation relates to the choice of an appropriate density threshold for binary-weighted graphs. To address this limitation, we monitored the ND values with a density range of 5–50% to allow for the trending of data. This range was found to be consistent with the biological background of brain functional networks^79–81^. The 5% lower bound was selected to avoid excessive network fragmentation at lower density, while the 50% upper bound was considerately chosen as the brain networks tend to move toward randomness at higher densities.

## Methods

### Animals

TgF344-AD rats were purchased from the RRRC and were bred in-house with wild-type F344 rats. Upon weaning of the litters, rats were genotyped to evaluate if the rats were expressing the human APP and PS1 gene, using the protocol provided by the RRRC. Next, male TgF344-AD rats (TG; *N* = 13), and wild-type male (WT; *N* = 11) littermates underwent longitudinal MRI at 4 and 6 months of age (named as TG_4M, WT_4M, and TG_6M, WT_6M, respectively). Animals were housed in pairs in an animal care facility with a 12 h light/dark cycle under controlled temperature (20–24 °C) and humidity (40–60%). Rats had access to standard food and water ad libitum. After the last scan session, animals were humanely sacrificed through an overdose of pentobarbital (intraperitoneally injected in Isoflurane anesthetized rats), followed by a transcardial perfusion with ice-cold PBS and 4% PFA. All procedures were in accordance with the guidelines of the European Ethics Committee (decree 2010/63/EU). The research was approved by the Committee on Animal Care and Use at the University of Antwerp, Belgium (approval number: 2019-06).

### rsfMRI acquisition

**Fig. 6:**
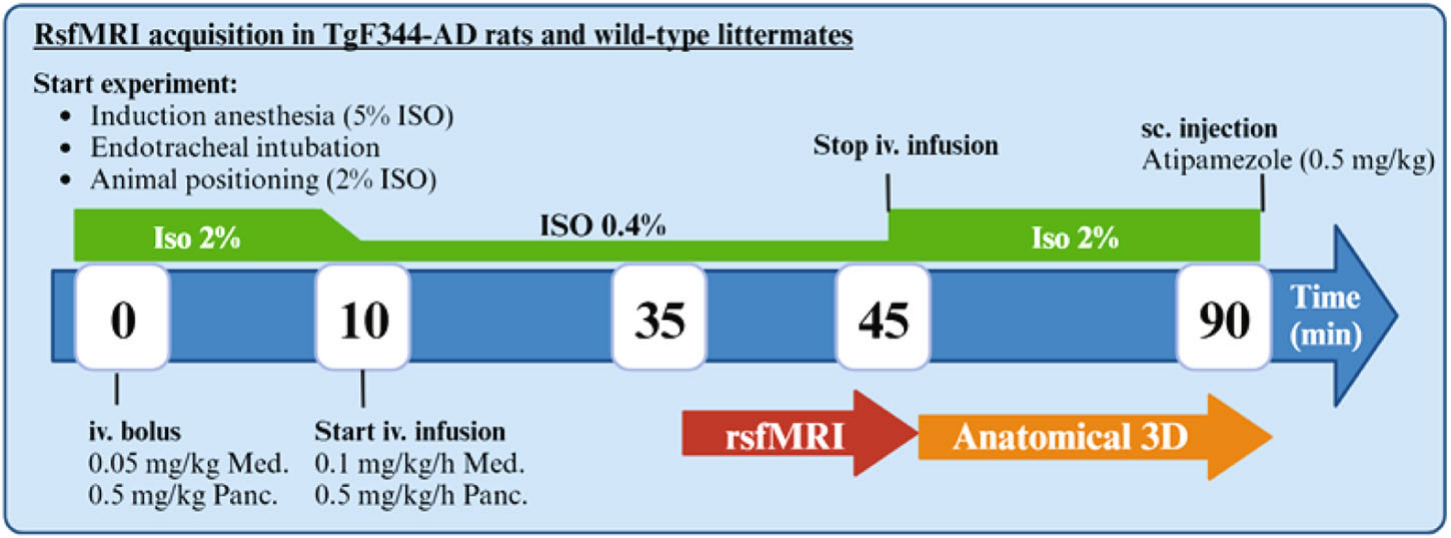
Schematic view of the data acquisition. TgF344-AD rats and age-matched wild-type littermates underwent rsfMRI. Anesthesia was induced with Isoflurane (ISO), endotracheally intubated and mechanically ventilated (70 breaths/min). After positioning of the animal in the scanner, intravenous (iv.) injection of medetomidine (Med) and Pancuroniumbromide (Panc) were administered, after which ISO was gradually lowered to 0.4%. A continuous infusion of Med and Panc was started ten minutes (min) after the bolus injection. Upon termination of the rsfMRI scan, ISO was increased to 2% and iv. Infusion was stopped. After the scan session, rats were subcutaneously (s.c.) injected with Atipamezole to counteract the effects of the anesthesia.

Figure 6 shows a schematic view of the data acquisition. Anesthesia was induced using isoflurane (5% for induction and 2% during handling procedures). The animals were endotracheally intubated and mechanically ventilated (70 breaths per minute) using a ventilator (Microventilator, Carfil, Belgium) with 2% isoflurane. Anesthesia during the resting state fMRI scan consisted of an intravenous bolus injection of medetomidine (0.05 mg/kg, Domitor®, Pfizer, Germany) and Pancuroniumbromide (0.5 mg/kg, VWR, Belgium), followed by infusion of medetomidine (0.1 mg/kg/hr) and Pancuroniumbromide (0.5 mg/kg/hr), along with 0.4% isoflurane. Body temperature was maintained at (37 ± 0.5) °C using a feedback-controlled warm air circuitry (MR-compatible Small Animal Heating System, SA Instruments, Inc., USA). Breathing rate, heart rate, and blood oxygenation were constantly monitored throughout the scan (MR-compatible Small Animal Monitoring and Gating System, SA Instruments, Inc., USA).

Data were acquired using a 9.4 T Biospec MRI system (Bruker BioSpin, Germany) with Paravision 6 software application. After slice positioning, local shimming with an ellipsoid volume was performed to correct for magnetic field inhomogeneity within the volume of interest. Resting-state functional MRI (rsfMRI) was acquired using a single-shot gradient-echo EPI-sequence with TR: 600 ms, TE: 18 ms, FOV: (30 × 30) mm^2^, imaging matrix: [96 × 96], 12 coronal slices: 12, slice thickness: 1.0 mm, and a number of repetitions: 1000, total scan duration was 10 min. In addition, T2-weighted 3D images were acquired using a 3D RARE sequence with TR: 2500 ms, TE: 44 ms, FOV: (30 x 30 x 22) mm^3^, imaging matrix: [256 x 256 x 128], and RARE-factor: 16, total scan duration 45 min. At the end of the scan, a subcutaneous injection of 0.1 mg/kg atipamezole (Antisedan®, Pfizer, Germany) was administered to counteract the effects of the medetomidine anesthesia. Then, Animals were mechanically ventilated on a second ventilator in a recovery box under an infrared light. Animals recovered within 1 h after the end of the scan. Two of the 4-month-old TgF344-AD rats did not recover after the scan due to premature extubation.

### fMRI analysis

**Fig. 7:**
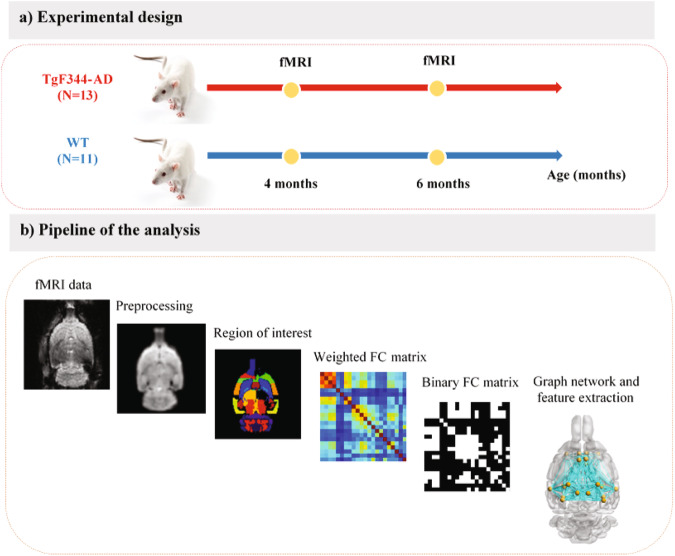
Experimental design and pipeline of the analyses. **a** Experimental design, (**b**) Pipeline of the analysis including: preprocessing, extracting functional connectivity matrix (FCM), thresholding to obtain the binary FCM, constructing graph network from binary FCM, extracting graph features (nodal degree), and running the statistical analyses.

The analysis consists of five major steps (Fig. 7b): (1) preprocessing, (2) extraction of the functional connectivity matrix (FCM) based on 25 regions of the Fischer344 atlas, (3) thresholding to obtain the binary FCM, (4) construction of the graph from the FCM and extracting graph features. including nodal degree, and (5) statistical analysis.

### Preprocessing

A series of standard pre-processing steps were performed on fMRI data using SPM12 software (Statistical Parametric Mapping), including: (i) 20 volumes (the first 10 and the last 10) were discarded to allow the signal to reach magnetization equilibrium and avoid edge effects due to filtering, (ii) debiasing on the 3D RARE scans in order to remove the smooth, spatially varying intensity gradient induced by the receiving RF coil, (iii) we created a study-specific 3D template using all 3D rare images from both groups at both timepoints using Advanced Normalization Tools (ANTS), (iv) a global 12-parameter affine transformation was used, followed by a nonlinear deformation protocol to create the study-based 3D template, (v) all individual 3D scans were spatially normalized to the study-specific 3D template using a global 12-parameter affine transformation followed by a non-linear deformation protocol, (vi) all rsfMRI images were realigned to the first image using a 6-parameter rigid body spatial transformation estimated using the least-square approach, (vii) rsfMRI images were co-registered to the anatomical 3D scan of the same imaging session using a global 12-parameter affine transformation with mutual information as the similarity metric, (viii) the co-registration parameters and the normalization parameters originating from the normalization from the 3D scans to the 3D template were applied to all rsfMRI images, (ix) white matter and ventricles signals were regressed on the rs-fMRI data, global signal was not regressed, (x) in-plane smoothing was performed using a Gaussian kernel with full width at half maximum (FWHM) of twice the voxel size, (xi) a temporal band-pass filter (0.01 < f < 0.17 Hz) was applied. Next, independent component analysis (ICA) was performed using the Infomax algorithm with a predefined number of 5 components on all subjects (both ages), to identify the regions which belong to the DMLN (GIFT toolbox, MATLAB).

### Construction of the DMLN graph from FCM and extracting ND centrality

We used the graph theory analysis to construct a brain functional network, utilizing Brain Analysis using Graph Theory (BRAPH) toolbox in MATLAB^82^. First, we examined the outcomes of the exploratory ICA to determine which regions of the DMLN should be included in the analysis, based on the presence of FC with other DMLN regions (refer to Fig. 1). For analysis of FC, 25 seed regions of the DMLN were chosen from the Fischer344 atlas (https://www.nearlab.xyz/fischer344atlas) based on the prior knowledge (Fig. 3). Specifically, the following brain regions were used in our analyses: left and right cingulate cortex (area1 and area2), left and right orbitofrontal cortex, left and right prelimbic cortex, left and right primary and secondary visual cortex, left and right retrosplenial cortex, left and right hippocampus CA sub regions (CA1, CA2, and CA3), left and right auditory/temporal association cortex, left and right parietal association cortex, and basal forebrain (in the Fischer344 atlas, the left and right basal forebrain are considered as a single region and labeled accordingly)^20,21,83^. For each subject, the BOLD signal time series for each region were extracted, then between each pair, the Pearson correlation was calculated to obtain a correlation matrix. Based on the correlation matrices, we constructed a weighted graph (weighted FCM) and Fisher’s r-to-z transformation was applied to improve normality for graph construction. The negative correlations were set to zero to improve the reliability of graph theoretical measures. A proportional thresholding method was applied to construct a binary functional connectivity matrix. This approach included assigning the value 1 to the strongest D% (density) of connections in each network and the value 0 to other connections^84^. To understand how ND changes as a function of density D, we threshold each FCM over a wide range of densities (5 ≤ D ≤ 50) at the interval of 1%. The threshold range analysis allows for a detailed exploration of connectivity patterns and network dynamics. The threshold range analysis highlights individual variability in connectivity patterns. Different individuals may have distinct threshold ranges where their connectivity patterns exhibit notable changes, providing insights into inter-individual differences. We calculated the ND values in the DMLN regions at each D% threshold and compared it between groups at each age. After applying each density threshold, the ND value was calculated as:

1 $${(ND)}_{i}={\sum }_{j\in N}{a}_{ij}$$

where N is the number of all nodes in the network, ${a}_{{ij}}$ is the connection value between a pair of nodes (i and j), with ${a}_{{ij}}=1$ when a connection between $(i,j)$ exists, and ${a}_{{ij}}=0$, otherwise^36,37^.

### Statistical analyses

To identify changes in the DMLN as reflected in the ND values, we performed comparisons across different conditions: 1) Genotype differences (TG vs WT) at 4 months of age (pre-plaque stage), 2) Genotype differences at 6 months of age (early plaque stage), and 3) the effect of age (4-month-old vs 6-month-old) in each group. For each comparison, we first calculated the mean nodal degree for individual area and condition and then computed their difference on an area-by-area basis per functional connectivity density (ranging from 5% - 50% in steps of 1%).

To identify if the differences in nodal degree were significant (*p* < 0.05, non-parametric permutation test, FDR corrected) we used a non-parametric permutation test. The permutation testing is a consented approach for controlling the nominal type I error. It is essentially used to determine whether the measured effect is genuine or is a statistical glitch due to the randomness associated with the sample selection^85^. BRAPH employs a nonparametric permutation test to assess the significance of the differences between cohorts (reported as *p*-values) and to determine the 95% confidence intervals^82^. Nonparametric permutation test with 10000 resamples was performed to evaluate the significance of differences in ND values between the TG and WT cohorts. In detail, the nonparametric permutation test (i) determines the difference between the measures in two cohorts, (ii (permutes the subjects across the two cohorts; calculates the difference between the measures in the permuted cohorts, repeats this procedure 10000 times, and obtains the histogram of the differences, (iii) determines whether the calculated difference between the two cohorts remains within the histogram, and (iv) compares the difference with a chosen threshold. To address multiple comparisons, the false discovery rate (FDR) algorithm was used at the *p* < 0.05 significance level^86^. The same procedure is applied to the longitudinal data analysis, with the distinction that only data corresponding to the same subject at different time points are permuted to prevent a group from containing data from multiple time points for the same subject.

### Reporting summary

Further information on research design is available in the Nature Research Reporting Summary linked to this article.

### Supplementary information

Supplementary

Reporting Summary

## Data Availability

Data can be made available upon reasonable request from corresponding authors.

## Code availability

This study did not involve the development of custom code or mathematical algorithms. The pre-processing steps for fMRI data utilized the open-source SPM12 software (Statistical Parametric Mapping), Advanced Normalization Tools (ANTS), and the GIFT toolbox in MATLAB. Following this, we applied graph theory analysis to construct a brain functional network, using the Brain Analysis using Graph Theory (BRAPH) toolbox in MATLAB. For information on access restrictions or licensing related to the codes, please look at the listed sources mentioned in the references.
