# Supplementary material for: Nodal degree centrality in the default mode-like network of the TgF344-AD Alzheimer’s disease rat model as a measure of early network alterations
Source: NPJ Aging. 2024 Jun 20;10(1):29. doi: 10.1038/s41514-024-00151-7 (PMC11190202; doi:10.1038/s41514-024-00151-7)
Supplement: Supplementary file 1 — Supplementary [file 41514_2024_151_MOESM1_ESM.pdf]

Supplementary Material

Supplementary Table 1 presents detailed information, such as p-values after FDR correction, for each density within different brain regions. This table helps to get a comprehensive understanding of the statistical significance in the studied regions.

Supplementary Table 1: The p-values after FDR correction in each density in brain regions: * and ** indicated p-value<0.05 and p-value<0.01, respectively. CA2: subregion of the hippocampus, prL: prelimbic Cortex, BFB: basal forebrain, CA1: subregion of the hippocampus, ptA: parietal association cortex, RS: retrosplenial.

**Density**	**CA2 (TG_4M vs. WT_4M)**	**PrL (TG_6M vs. WT_6M)**	**BFB (TG_6M vs. WT_6M)**	**CA1 (TG_6M vs. TG_4M)**	**PtA (TG_6M vs. TG_4M)**	**PrL (WT_6M vs. WT_4M)**	**RS (WT_6M vs. WT_4M)**
**5**	0.34	0.015*	0.31	0.18	0.25	0.028*	0.0001**
**6**	0.23	0.014*	0.31	0.21	0.37	0.011*	0.0001**
**7**	0.25	0.062	0.22	0.16	0.14	0.023*	0.0039**
**8**	0.32	0.045*	0.25	0.14	0.092	0.0136*	0.013*
**9**	0.25	0.019*	0.32	0.15	0.097	0.0084**	0.0085**
**10**	0.28	0.018*	0.24	0.093	0.11	0.011*	0.044*
**11**	0.2	0.013*	0.17	0.098	0.052	0.0022**	0.031*
**12**	0.33	0.01*	0.075	0.0808	0.057	0.0018**	0.056
**13**	0.38	0.02*	0.023*	0.084	0.11	0.0045**	0.077
**14**	0.26	0.033*	0.021*	0.056	0.081	0.0055**	0.048*
**15**	0.16	0.031*	0.0182*	0.061	0.095	0.0077**	0.061
**16**	0.21	0.035*	0.015*	0.069	0.078	0.013*	0.07
**17**	0.12	0.071	0.008**	0.089	0.025*	0.017*	0.055
**18**	0.10	0.042*	0.0159*	0.09	0.037*	0.0135*	0.062
**19**	0.06	0.055	0.0076**	0.096	0.043*	0.035*	0.043*
**20**	0.07	0.043*	0.0121*	0.12	0.037*	0.018*	0.021*
**21**	0.10	0.05*	0.0129*	0.091	0.027*	0.0126*	0.012*
**22**	0.056	0.056	0.0186*	0.078	0.038*	0.016*	0.0064**
**23**	0.056	0.021*	0.032*	0.071	0.039*	0.0044**	0.0145*
**24**	0.009**	0.0087**	0.031*	0.068	0.025*	0.004**	0.004**
**25**	0.026*	0.0088**	0.03*	0.047*	0.021*	0.0055**	0.0085**
**26**	0.004**	0.0038**	0.042*	0.042*	0.005**	0.0001**	0.0053**
**27**	0.02*	0.0083**	0.025*	0.045*	0.009**	0.0014**	0.01*
**28**	0.042*	0.0024**	0.028*	0.046*	0.009**	0.0001**	0.0075**
**29**	0.03*	0.0052**	0.029*	0.072	0.009**	0.0019**	0.0018**
**30**	0.02*	0.0025**	0.034*	0.062	0.023*	0.0009**	0.0011**
**31**	0.006**	0.0019**	0.042*	0.059	0.02*	0.003**	0.0123*
**32**	0.002**	0.0056**	0.035*	0.041*	0.042*	0.0023**	0.0185*
**33**	0.045*	0.0042**	0.033*	0.054	0.041*	0.0029**	0.0217*

**34**	0.054*	0.0055**	0.058	0.058	0.048*	0.001**	0.0425*
**35**	0.035*	0.01*	0.073	0.062	0.039*	0.0024**	0.041*
**36**	0.049*	0.014*	0.066	0.059	0.041*	0.0033**	0.11
**37**	0.049*	0.02*	0.081	0.059	0.037*	0.0033**	0.0385*
**38**	0.067	0.013*	0.088	0.049*	0.032*	0.0007**	0.029*
**39**	0.054	0.012*	0.074	0.035*	0.026*	0.0009**	0.0098**
**40**	0.026*	0.016*	0.097	0.028*	0.014*	0.0063**	0.0098**
**41**	0.029*	0.015*	0.088	0.031*	0.022*	0.0073**	0.0073**
**42**	0.051	0.017*	0.061	0.023*	0.03*	0.035*	0.02*
**43**	0.088	0.022*	0.051	0.009**	0.042*	0.083	0.059
**44**	0.13	0.029*	0.056	0.008**	0.046*	0.11	0.065
**45**	0.13	0.035*	0.075	0.0061**	0.026*	0.07	0.11
**46**	0.16	0.045*	0.1	0.0053**	0.031*	0.11	0.11
**47**	0.2	0.035*	0.115	0.0041**	0.042*	0.115	0.063
**48**	0.18	0.035*	0.11	0.0016**	0.033*	0.093	0.035*
**49**	0.27	0.043*	0.095	0.0009**	0.0416*	0.094	0.059
**50**	0.24	0.051	0.095	0.0017**	0.025*	0.147	0.037*
