# Supplementary material for: Nodal degree centrality in the default mode-like network of the TgF344-AD Alzheimer’s disease rat model as a measure of early network alterations
Source: NPJ Aging. 2024 Jun 20;10(1):29. doi: 10.1038/s41514-024-00151-7 (PMC11190202; doi:10.1038/s41514-024-00151-7)
Supplement: Supplementary file 2 — Reporting Summary [file 41514_2024_151_MOESM2_ESM.pdf]

nature portfolio

Corresponding author(s): Mahmood amiri

Last updated by author(s): YYYY-MM-DD

Reporting Summary

Nature Portfolio wishes to improve the reproducibility of the work that we publish. This form provides structure for consistency and transparency in reporting. For further information on Nature Portfolio policies, see our Editorial Policies and the Editorial Policy Checklist.

Statistics

For all statistical analyses, confirm that the following items are present in the figure legend, table legend, main text, or Methods section.

n/a	Confirmed	
☐	☒	The exact sample size (*n*) for each experimental group/condition, given as a discrete number and unit of measurement
☐	☒	A statement on whether measurements were taken from distinct samples or whether the same sample was measured repeatedly
☐	☒	The statistical test(s) used AND whether they are one- or two-sided<br>*Only common tests should be described solely by name; describe more complex techniques in the Methods section.*
☐	☒	A description of all covariates tested
☐	☒	A description of any assumptions or corrections, such as tests of normality and adjustment for multiple comparisons
☐	☒	A full description of the statistical parameters including central tendency (e.g. means) or other basic estimates (e.g. regression coefficient) AND variation (e.g. standard deviation) or associated estimates of uncertainty (e.g. confidence intervals)
☐	☒	For null hypothesis testing, the test statistic (e.g. *F*, *t*, *r*) with confidence intervals, effect sizes, degrees of freedom and *P* value noted<br>*Give P values as exact values whenever suitable.*
☒	☐	For Bayesian analysis, information on the choice of priors and Markov chain Monte Carlo settings
☒	☐	For hierarchical and complex designs, identification of the appropriate level for tests and full reporting of outcomes
☒	☐	Estimates of effect sizes (e.g. Cohen's *d*, Pearson's *r*), indicating how they were calculated

Our web collection on statistics for biologists contains articles on many of the points above.

Software and code

Policy information about availability of computer code

Data collection	The data were acquired using the Bruker Paravision 6.01 software platform.
Data analysis	This study did not involve the development of custom code or mathematical algorithms. The pre-processing steps for fMRI data utilized the open-source SPM12 software (Statistical Parametric Mapping), Advanced Normalization Tools (ANTS), and the GIFT toolbox in MATLAB. Following this, we applied graph theory analysis to construct a brain functional network, using the Brain Analysis using Graph Theory (BRAPH) toolbox in MATLAB. For information on access restrictions or licensing related to the codes, please look at the listed sources mentioned in the references.

For manuscripts utilizing custom algorithms or software that are central to the research but not yet described in published literature, software must be made available to editors and reviewers. We strongly encourage code deposition in a community repository (e.g. GitHub). See the Nature Portfolio guidelines for submitting code & software for further information.

Data

Policy information about availability of data

All manuscripts must include a data availability statement. This statement should provide the following information, where applicable:

- Accession codes, unique identifiers, or web links for publicly available datasets
- A description of any restrictions on data availability
- For clinical datasets or third party data, please ensure that the statement adheres to our policy

Data can be made available upon reasonable request.

Research involving human participants, their data, or biological material

Policy information about studies with human participants or human data. See also policy information about sex, gender (identity/presentation), and sexual orientation and race, ethnicity and racism.

Reporting on sex and gender	*Use the terms sex (biological attribute) and gender (shaped by social and cultural circumstances) carefully in order to avoid confusing both terms. Indicate if findings apply to only one sex or gender; describe whether sex and gender were considered in study design; whether sex and/or gender was determined based on self-reporting or assigned and methods used. Provide in the source data disaggregated sex and gender data, where this information has been collected, and if consent has been obtained for sharing of individual-level data; provide overall numbers in this Reporting Summary. Please state if this information has not been collected. Report sex- and gender-based analyses where performed, justify reasons for lack of sex- and gender-based analysis.*
Reporting on race, ethnicity, or other socially relevant groupings	*Please specify the socially constructed or socially relevant categorization variable(s) used in your manuscript and explain why they were used. Please note that such variables should not be used as proxies for other socially constructed/relevant variables (for example, race or ethnicity should not be used as a proxy for socioeconomic status). Provide clear definitions of the relevant terms used, how they were provided (by the participants/respondents, the researchers, or third parties), and the method(s) used to classify people into the different categories (e.g. self-report, census or administrative data, social media data, etc.) Please provide details about how you controlled for confounding variables in your analyses.*
Population characteristics	*Describe the covariate-relevant population characteristics of the human research participants (e.g. age, genotypic information, past and current diagnosis and treatment categories). If you filled out the behavioural & social sciences study design questions and have nothing to add here, write "See above."*
Recruitment	*Describe how participants were recruited. Outline any potential self-selection bias or other biases that may be present and how these are likely to impact results.*
Ethics oversight	*Identify the organization(s) that approved the study protocol.*

Note that full information on the approval of the study protocol must also be provided in the manuscript.

Field-specific reporting

Please select the one below that is the best fit for your research. If you are not sure, read the appropriate sections before making your selection.

☒ Life sciences ☐ Behavioural & social sciences ☐ Ecological, evolutionary & environmental sciences

For a reference copy of the document with all sections, see nature.com/documents/nr-reporting-summary-flat.pdf

Life sciences study design

All studies must disclose on these points even when the disclosure is negative.

Sample size	To estimate the sample size we used power analysis in the G*Power software where the variability was estimated from previous experiments.
Data exclusions	*Describe any data exclusions. If no data were excluded from the analyses, state so OR if data were excluded, describe the exclusions and the rationale behind them, indicating whether exclusion criteria were pre-established.*
Replication	*Describe the measures taken to verify the reproducibility of the experimental findings. If all attempts at replication were successful, confirm this OR if there are any findings that were not replicated or cannot be reproduced, note this and describe why.*
Randomization	The animals were allocated to experimental groups according to their genotype but this was only revealed after acquisition and analysis.
Blinding	The investigators were blinded in regard to the group the animals belonged by using subject IDs that were independent of the group

Reporting for specific materials, systems and methods

We require information from authors about some types of materials, experimental systems and methods used in many studies. Here, indicate whether each material, system or method listed is relevant to your study. If you are not sure if a list item applies to your research, read the appropriate section before selecting a response.

Materials & experimental systems

n/a	Involved in the study
☒	☐ Antibodies
☒	☐ Eukaryotic cell lines
☒	☐ Palaeontology and archaeology
☐	☒ Animals and other organisms
☒	☐ Clinical data
☒	☐ Dual use research of concern
☒	☐ Plants

Methods

n/a	Involved in the study
☒	☐ ChIP-seq
☒	☐ Flow cytometry
☐	☒ MRI-based neuroimaging

Animals and other research organisms

Policy information about studies involving animals; ARRIVE guidelines recommended for reporting animal research, and Sex and Gender in Research

Laboratory animals	TgF344-AD (rat model of Alzheimer's Disease) and wild-type littermates
Wild animals	N/A
Reporting on sex	Only male animals were used in this study because of feasibility reasons and to limit variability and sample size
Field-collected samples	N/A
Ethics oversight	The research was approved by the Committee on Animal Care and Use at the University of Antwerp, Belgium (approval number: 2019-06)

Note that full information on the approval of the study protocol must also be provided in the manuscript.

Plants

Seed stocks	*Report on the source of all seed stocks or other plant material used. If applicable, state the seed stock centre and catalogue number. If plant specimens were collected from the field, describe the collection location, date and sampling procedures.*
Novel plant genotypes	*Describe the methods by which all novel plant genotypes were produced. This includes those generated by transgenic approaches, gene editing, chemical/radiation-based mutagenesis and hybridization. For transgenic lines, describe the transformation method, the number of independent lines analyzed and the generation upon which experiments were performed. For gene-edited lines, describe the editor used, the endogenous sequence targeted for editing, the targeting guide RNA sequence (if applicable) and how the editor was applied.*
Authentication	*Describe any authentication procedures for each seed stock used or novel genotype generated. Describe any experiments used to assess the effect of a mutation and, where applicable, how potential secondary effects (e.g. second site T-DNA insertions, mosiacism, off-target gene editing) were examined.*

Magnetic resonance imaging

Experimental design

Design type	Resting state fMRI
Design specifications	A single EPI scan with the duration of 10 minutes and an anatomical scan with a duration of 45 minutes for each subject.
Behavioral performance measures	N/A

Acquisition

Imaging type(s)	Functional and Anatomical MRI
Field strength	9.4 T
Sequence & imaging parameters	Data were acquired using a 9.4T Biospec MRI system (Bruker BioSpin, Germany) with Paravision 6 software application. After slice positioning, local shimming with an ellipsoid volume was performed to correct for magnetic field inhomogeneity within the volume of interest. Resting-state functional MRI (rsfMRI) was acquired using a single-shot gradient-echo EPI-sequence with TR: 600ms, TE: 18ms, FOV: (30 x 30) mm2, imaging matrix: [96 x 96], 12 coronal slices: 12, slice thickness: 1.0 mm, and a number of repetitions: 1000, total scan duration was 10 minutes. In addition, T2-weighted 3D images were acquired using a 3D RARE sequence with TR: 2500 ms, TE: 44 ms, FOV: (30x30x22) mm3, imaging matrix: [256x256x128], and RARE-factor: 16, total scan duration 45 minutes. At the end of the scan, a subcutaneous injection of 0.1 mg/kg atipamezole (Antisedan®, Pfizer, Germany) was administered to counteract the effects of the medetomidine anesthesia.
Area of acquisition	whole brain

Diffusion MRI ☐ Used ☒ Not used

Preprocessing

Preprocessing software	SPM12, ANTS, GIFT
Normalization	Each subject was normalized to a template atlas
Normalization template	Fischer344 atlas (https://www.nearlab.xyz/fischer344atlas)
Noise and artifact removal	*Describe your procedure(s) for artifact and structured noise removal, specifying motion parameters, tissue signals and physiological signals (heart rate, respiration).*
Volume censoring	*Define your software and/or method and criteria for volume censoring, and state the extent of such censoring.*

Statistical modeling & inference

Model type and settings	We used a non-parametric permutation test.
Effect(s) tested	*Define precise effect in terms of the task or stimulus conditions instead of psychological concepts and indicate whether ANOVA or factorial designs were used.*

Specify type of analysis: ☐ Whole brain ☒ ROI-based ☐ Both

Anatomical location(s)	25 seed regions of the DMLN were chosen from the Fischer344 atlas (https://www.nearlab.xyz/fischer344atlas)
Statistic type for inference (See Eklund et al. 2016)	Voxel - wise
Correction	To address multiple comparisons, the false discovery rate (FDR) algorithm was used at the p<0.05 significance level.

Models & analysis

n/a	Involved in the study
☐	☒ Functional and/or effective connectivity
☐	☒ Graph analysis
☒	☐ Multivariate modeling or predictive analysis

Functional and/or effective connectivity	The Pearson correlation coefficient was used to calculate the functional connectivity matrix
Graph analysis	We computed the nodal degree values in the binarized graph at the group level.
